# The effects of different positions on lower extremity hemodynamics during robot-assisted laparoscopic radical prostatectomy for prostate cancer

**DOI:** 10.1186/s12894-024-01462-3

**Published:** 2024-04-20

**Authors:** Zheng Wang, Xinyu Wang, Xiaofen Yu

**Affiliations:** 1 Cancer Center, Gamma Knife Treatment Center, Zhejiang Provincial People’s Hospital, Affiliated People’s Hospital, Hangzhou Medical College, Hangzhou, Zhejiang China; 2 Graduated School, Zhejiang Chinese Medical University, Hangzhou, 310014 Zhejiang China; 3 Urology & Nephrology Center, Department of Nursing, Zhejiang Provincial People’s Hospital, Affiliated People’s Hospital, Hangzhou Medical College, Hangzhou, 310014 Zhejiang China; 4 Nursing Department, Zhejiang Provincial People’s Hospital, Affiliated People’s Hospital, Hangzhou Medical College, Hangzhou, 310014 Zhejiang China

**Keywords:** Robot-assisted laparoscopic radical prostatectomy, Position, Deep vein, Lower extremity, Hemodynamics

## Abstract

**Purpose:**

This study aimed to investigate the effects of two different positions on lower extremity hemodynamics during robot-assisted laparoscopic radical prostatectomy (RARP) for prostate cancer.

**Methods:**

A total of 196 patients who underwent RARP in our hospital from February 2020 to March 2022 were included in this study. Among them, 98 patients who underwent surgery with the Trendelenburg position and split-leg position with calf reverse arch from March 2021 to March 2022 were assigned to the observation group, while 98 patients who underwent surgery with the Trendelenburg position and low lithotomy position from February 2020 to February 2021 were assigned to the control group. Using an ultrasound diagnostic instrument to detect the internal diameter, mean blood flow velocity, and mean blood flow volume of the left deep femoral vein at different times, such as the supine position (T0), after 5 minutes of placing the patient in the leg spilt or low lithotomy position (T1), after 5 minutes of pneumoperitoneum (T2), after 5 minutes of head-down tilt or head-down tilt and calf reverse arch (T3), 1.5 hours after the start of surgery (T4), before the removal of CO2 gas (T5), and before the patient left the operating room (T6). As well as the patency of deep venous blood flow in both lower extremities before leaving the operating room,

**Results:**

After establishment of pneumoperitoneum, the internal diameter of the deep femoral vein increased significantly, while the mean blood flow velocity and mean blood flow volume decreased significantly in both groups(T0) (*P*<0.001). With the prolongation of surgical time, the impact on lower extremity hemodynamics in the observation group was smaller than that in the control group. From T2 to T6, the internal diameter of the femoral vein in the observation group was smaller than that in the control group, while the mean blood flow velocity and mean blood flow volume were increased compared to the control group (*P*<0.05). Before leaving the operating room, the patency of deep venous blood flow in the observation group was better than that in the control group (*P*=0.003).

**Conclusion:**

Placing patients in the Trendelenburg position and split-leg position with calf reverse arch during RARP for prostate cancer has a smaller impact on lower extremity hemodynamics than the low lithotomy position, and can relatively reduce the risk of postoperative deep vein thrombosis.

## Introduction

The Da Vinci surgical system (DVSS) is a robotic surgical system that utilizes a 3D high-definition visualization system to expand the surgical field of view, overcoming the limitations of the traditional laparoscopic flat view and providing a clearer and more realistic view of the surgical site. The multi-directional simulation mechanical wrist operation and the filtering of the surgeon's hand tremors compensate for the deficiency of traditional laparoscopic instruments in terms of dexterity, making the operation more stable, precise, and facilitating the protection of important anatomical structures and the reconstruction of organ physiological functions. The system has been widely used in urological surgery [1, 2].

Robot-assisted laparoscopic radical prostatectomy (RARP) is performed using the Si Da Vinci surgical system. The patient is placed on a movable platform, which is inserted through the patient's posterior end. The patient's hip joint needs to be externally rotated at an angle of 50°-60° to fully utilize gravity and expose the potential inter-organ and inter-space cavities. During the operation, the head of the operating table should be lowered and the feet raised to the maximum extent to adjust the inclination angle of the operating bed.

During the RARP operation, various factors, such as carbon dioxide pneumoperitoneum, head-down tilt position, surgical operations, anesthesia, disease nature, and the age of the patient, may increase blood viscosity, damage the venous endothelium, and delay venous blood flow in the lower limbs. The external rotation of the hip joint during the operation may cause the femoral vein to be pulled or compressed, resulting in a decrease in the inner diameter of the femoral vein and further delaying venous blood flow in the lower limbs, thereby increasing the risk of deep vein thrombosis (DVT) [3]. DVT is the main source of secondary pulmonary thromboembolism (PTE) and can lead to a poor prognosis if left untreated. Therefore, taking active comprehensive preventive measures is of utmost importance [4].

Our robotic surgery team has consulted relevant literature [5–7] and combined clinical practice to continuously improve the intraoperative position of RARP, maintaining the hip joint function and ensuring patient safety and maximum exposure of the surgical field. This study compares the hemodynamics of two commonly used positions in RARP and provides feasibility and practicality for its clinical application.

## Information and methods

### Clinical information

Prospective clinical data from a study conducted in a tertiary hospital in Zhejiang Province, China, from February 2020 to March 2022, were used to investigate 196 patients who underwent robot-assisted radical prostatectomy (RARP) within the same medical group. All surgeries were performed by a dedicated team of nurses. All experiments were carried out in accordance with relevant guidelines and regulations, and this project was approved by the ethics committee of Zhejiang Provincial People's Hospital (2022009), and informed consent was obtained from all subjects.

### Inclusion/exclusion criteria

Inclusion criteria were as follows: 1) patients had a confirmed pathological diagnosis from a prostate biopsy before surgery, with no evidence of distant metastasis on imaging examinations; 2) patients had complete surgical video data, were conscious and able to communicate effectively, had an American Society of Anesthesiologists (ASA) classification of I to II, and did not experience hypothermia during the operation; 3) patients had no history of abdominal or pelvic surgery, radiation therapy (except for prostate biopsy), neoadjuvant chemotherapy, severe chronic diseases, or difficulty tolerating carbon dioxide pneumoperitoneum; and 4) patients had not used drugs affecting the coagulation or fibrinolysis systems in the 2 weeks before surgery, and ultrasound examination of the lower limbs showed unobstructed venous blood flow before anesthesia. Exclusion criteria were as follows: 1) patients with a history of venous thromboembolism (VTE) who had received anticoagulant or thrombolytic therapy before surgery; 2) patients with cardiovascular diseases that were prone to thrombosis, such as atrial fibrillation, congestive heart failure, or pulmonary edema, or other concomitant malignant tumors; 3) patients with abnormal lower limb local conditions, including swelling, necrosis, dermatitis, skin graft surgery, severe limb deformity, joint surgery history, or ischemic vascular disease, such as arterial sclerosis; and 4) patients with a body mass index (BMI) of ≥30 kg/m2. Additional exclusion criteria were as follows: 1) changing the surgical approach to laparoscopy or open surgery during the operation, and 2) transfusion of blood products during the operation.

## Grouping program

### Observation group

#### Positioning equipment

An angle measuring tool, a 45cm × 25cm × 12cm sponge pillow, a gel buttock pad, two restraint straps, and two cotton pads.

#### Positioning method

**Fig. 1:**
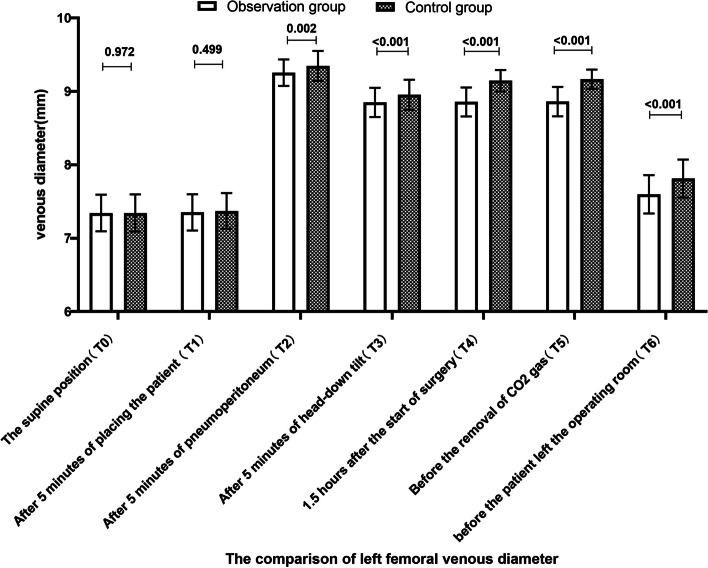
Positions at different times in two groups. Observation group: **a** supine position; **b** split-leg position; **c** Trendelenburg position and split-leg position with calf reverse arch; control group: **d** supine position; **e** low lithotomy position; **f** Trendelenburg position and low lithotomy position

(1) Supine position (Fig. 1a): Place a 45cm × 25cm × 12cm sponge pillow under the patient's neck, and place the knee joint at the junction of the surgical table leg board and back board. The rest is the same as the control group; (2) Split-leg position (Fig. 1b): On the basis of the supine position, abduct the left and right leg boards in parallel by 50° to 60°, fix the restraint straps 3-5 cm below the knee joint, and place cotton pads at the popliteal fossa and the restraint strap skin contact. Perform general anesthesia and establish pneumoperitoneum; (3) Trendelenburg position and split-leg position with calf reverse arch (Fig. 1c): Adjust the surgical table to a head-down position of 25°-28° before docking the robot, raise the back panel by 10°-15°, adjust the lower leg board down so that the knee joint is flexed at 120° to 130°. The thigh remains horizontal, the lower leg naturally hangs down, and the distal end of the lower limb, the foot, is at the same level as the heart, forming a head-down position with calf hanging split-leg position.

### Control group

#### Positioning equipment

An angle measuring tool, a 4cm × 20cm × 6cm sponge pillow, a gel coccyx pad, shoulder pads, shoulder pad fixators, two saddle-shaped multifunctional leg supports (OTJ-001, Tianjin), and two fixators, and two cotton pads.

#### Positioning method

(1) Supine position (Fig. 1d): Place a sponge pillow under the patient's head, with the head and cervical spine in a neutral position, a gel coccyx pad under the sacrum, and the patient's thighs even with the edge of the surgical table back panel. The upper limbs are naturally placed on both sides of the body, palms facing each other, and fixed to the top layer of the surgical table with a single sheet previously laid; (2) Low lithotomy position (Fig. 1e): On the basis of the supine position, place the saddle-shaped multifunctional leg supports on the near hip joint plane, with the leg supports level with the surgical table. Adjust the angle of the leg rest to the patient's comfort, abduct both lower limbs by 60° to 70°, and flex the knee joint slightly to the patient's comfort. After fixing the knee joint with a restraint strap 3-5 cm below the joint, perform general anesthesia, and fix the patient's shoulders with shoulder pads and establish pneumoperitoneum;(3) Trendelenburg position in low lithotomy position (Fig. 1f): Adjust the surgical table to a head-down position of 25°-28° before docking the robot, and raise the back panel by 10°-15°.

### Safety management

(1) Before positioning the patient, assess the range of motion of the lower limbs and check for a history of high intraocular pressure such as glaucoma. Ensure that the operating table is in good working condition. (2) During positioning, ensure warmth and privacy protection. Adjust the insufflation pressure to 12 mmHg (1 mmHg = 0.133 kPa) before insufflation. The flow rate should start at a low speed of 1 L/min, and the position of the insufflation needle should be checked based on the speed of the gas entering and the rate of abdominal pressure increase. Once the position is correct, adjust the flow rate to medium speed (1-20 L/min). The intra-abdominal pressure should be maintained at 12-14 mmHg and not exceed 15 mmHg during the operation. (3) After positioning, use a goniometer to accurately measure the angle of the head and leg board of the operating table to ensure that the position is correct. (4) Comprehensive warming measures should be taken during the operation [8]. (5) When it does not affect the surgeon's operation, the lower limbs should be massaged regularly, and the bedside platform should be moved forward and backward. The circulating nurse should confirm the safety of the positioning. When moving the platform, avoid crushing the patient's skin with the base of the platform, mechanical arm, or lens arm, and adjust the height of the anesthesia frame to avoid affecting the range of motion of the lens arm. After closing the patient's upper and lower eyelids, apply infusion tape to protect the eyes and use cotton pads to protect the head and face. (6) When the sterile barrier is established, avoid contaminating the surgical area when measuring hemodynamic indicators.

### Anesthesia program

General anesthesia is used in both groups. Oxygen is given by facemask, then Patients are injected intravenously with midazolam at 0.05~0.1mg/Kg, propofol at 1.5~2.5mg/Kg and fentanyl at l4g/Kg. And muscle relaxation is induced with cis-atracurium bolus (0.15mg/Kg). Mechanical ventilation was started after tracheal tube placement. The ventilator set to Intermittent Positive Ventilation(IPPV), the tidal volume is 8-10ml/Kg, the respiratory rate is 12-14 breaths/min. the inspiration and expiration ratio(I/E) is 1/2. The P_ET_CO_2_ is controlled in the range of 35-40 mmHg. Regulate FiO2 to maintain SpO2 >96%. In the operation, the drugs are adjusted according to blood pressure and bispectral index(BIS). Fluctuate blood pressure within pre-operative ±20%.

## Detection method

### The time to detect

A diagnostic ultrasound examination was used to sequentially detect the diameter (D) and blood flow velocity (V) of the left deep femoral vein in two groups of patients in the supine position (T0), after 5 minutes of placing the patient in the leg spilt or low lithotomy position (T1), after 5 minutes of pneumoperitoneum (T2), after 5 minutes of head-down tilt or head-down tilt and calf reverse arch (T3), 1.5 hours after the start of surgery (T4), before the removal of CO2 gas (T5), and before the patient left the operating room (T6). The formula Q=Vπ(D/2)2 was used to calculate the blood flow rate (Q) in the left deep femoral vein per unit time.

Before the patients left the operating room, the diagnostic ultrasound examination was performed on their lower extremities to assess venous blood flow. The ultrasound results were assessed by a radiologist and recorded as normal, stagnant, or indicative of deep vein thrombosis (DVT).

### The detail of the ultrasound examination

Use an ultrasound diagnostic device produced by the American ATL company (HDIll000) with a 4.0 MHz ultrasound probe. Place the probe of the common femoral vein where it joins the deep femoral vein, which is approximately 3.5 cm below the inguinal ligament and lateral to the pubic tubercle. Use a 2D ultrasound cross-section to measure the diameter (D) of the left deep femoral vein. Adjust the probe to the specified orientation (with a blood flow angle of 52°), and use pulsed wave Doppler to measure the blood flow velocity (V) in the deep femoral vein. The examination should be performed by a physician with more than 5 years of experience in ultrasound diagnosis.

## Statistical methods

The collected data was organized and analyzed using Epidata 3.1 to establish a database and SPSS 26.0 statistical software was used for data analysis. Frequency and composition ratios were used to describe count data, while rates were used for statistical comparison using the chi-square (χ2) test. Normally distributed metric data was described using the mean ± standard deviation (¯χ±s), and intergroup comparisons were made using the t-test. Skewed metric data was described using the median and quartiles [M (P25, P75)], and intergroup comparisons were made using the Mann-Whitney U test. The Kruskal-Wallis rank-sum test was used for within-group comparisons, and Bonferroni's test was used for multiple comparisons. A *P* value of less than 0.05 was considered statistically significant.

## Results

### The general data of patients

The observation group consisted of 98 consecutive patients from March 2021 to March 2022, who were sequentially placed in supine, split-leg, and Trendelenburg positions with calf reverse arch; the control group consisted of 98 patients from February 2020 to February 2021, who were sequentially placed in supine, low lithotomy, and Trendelenburg positions. There were no statistically significant differences in basic patient information between the two groups (*P* > 0.05), indicating comparability, as shown in Table 1.

**Table 1:** Comparison of general data between the two groups

Group	Age (Years, _x±s)		BMI [kg/m2, M(P25, P75)]	Prostate volume [cm^3^, M(P25, P75)]		Preoperative PSA (ng/ml, _x±s)	ASA (cases, %)	
							Grade I	Grade II
Observation group (*n*=98)	66.92±4.68		23.50(22.60,24.70)	43.00(42.00,46.00)		13.20(9.68,18.43)	36(36.73)	62(63.27)
Control group (*n*=98)	66.50±5.16		23.30(22.60,24.30)	43.00(41.00,45.25)		13.80(8.68,18.68)	34(34.69)	64(65.31)
*t/Z/χ2* value	t=0.595		Z=0.504	Z=1.075		Z=0.251	χ2=0.089	
*P* value	0.553		0.614	0.283		0.802	0.766	
Group	Risk of Caprini thrombosis (cases, %)		Preoperative TNM staging (cases, %)				Lymph node dissection (cases, %)	
	High risk	Extremely high risk	cT1c	cT2a	cT2b	cT2c	Yes	No
Observation group (n=98)	34(34.69)	64(65.31)	3(3.06)	31(31.63)	40(40.82)	24(24.49)	23(23.47)	75(76.53)
Control group (n=98)	32(32.65)	66(67.35)	4(4.08)	30(30.61)	37(37.76)	27(27.55)	19(19.39)	79(80.61)
χ2 value	0.091		3.059				0.710	
*P* value	0.762		0.801				0.701	

(1) *PSA* Prostate specific antigen

(2) Caprini thrombus risk was assessed according to the guidelines for diagnosis and treatment of deep vein thrombosis (the third edition of 2017) [7]: low risk: 0 to 2 points; Moderate risk: 3-4 points; High risk: ≥ 5 points

### Comparison of left deep femoral vein diameter, mean blood flow velocity, and mean blood flow rate in different positions between two groups

**Fig. 2:**
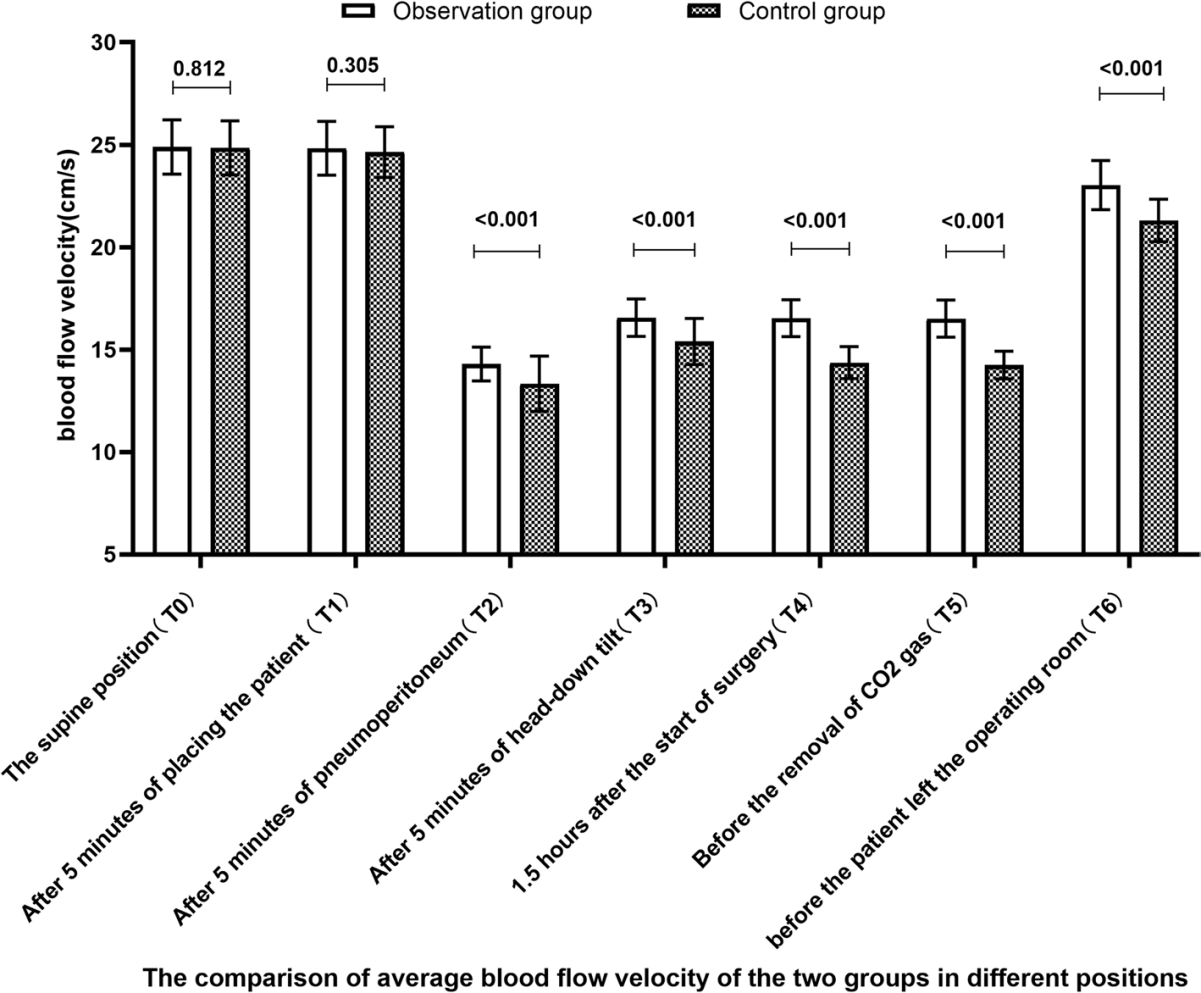
The comparison of deep left femoral venous diameter of the two groups in different positions [mm, M (P25, P75)]

**Fig. 3:**
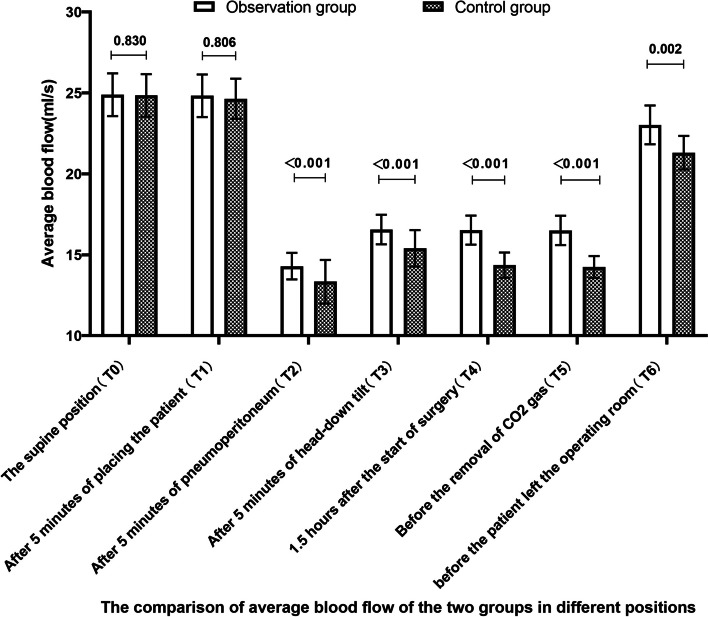
The comparison of average blood flow velocity of the two groups in different positions [(cm/s, $\overline{\upchi }$±s), M (P25, P75)]

**Fig. 4:**
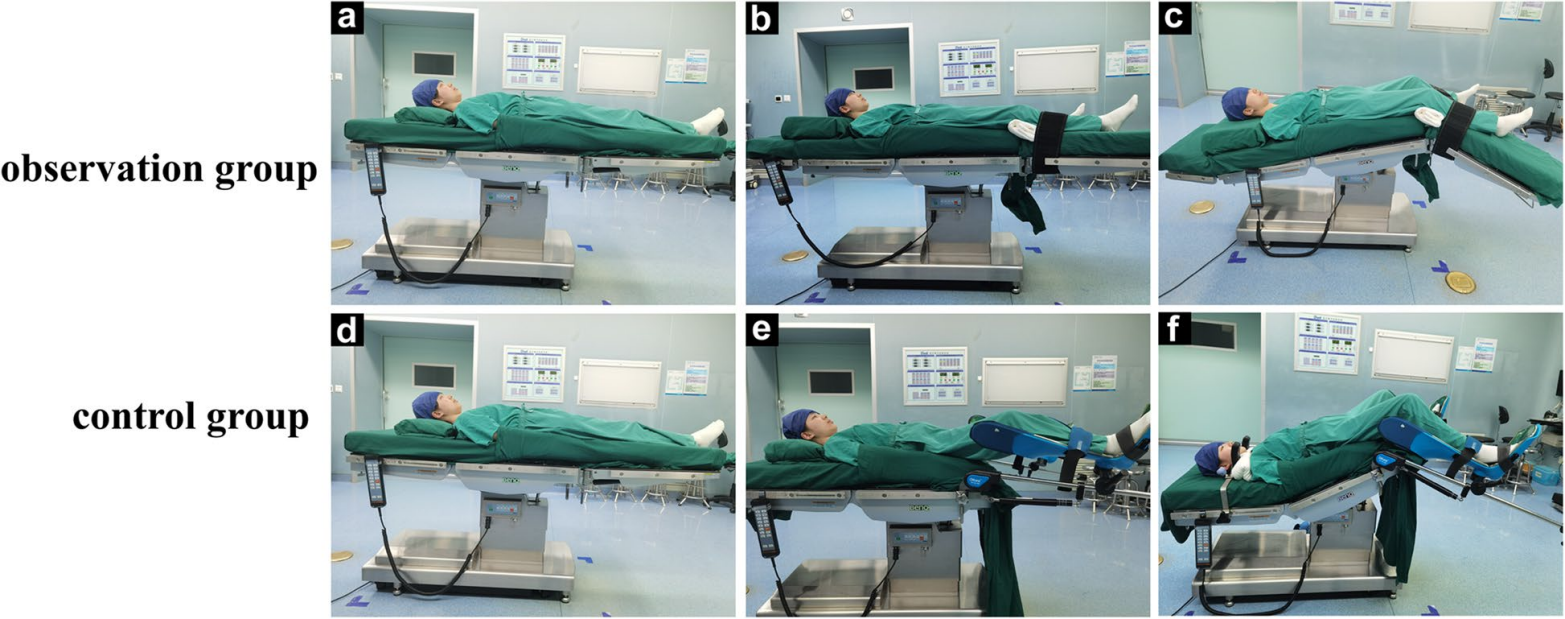
The comparison of average blood flow of the two groups in different positions [(ml/s, $\overline{\upchi }$±s), M (P25, P75)]

The results can be seen in Figs. 2, 3 and 4. After pneumoperitoneum establishment, the deep femoral vein diameter significantly increased, and the mean blood flow velocity and mean blood flow rate significantly decreased in both groups, with statistically significant differences compared to pre-pneumoperitoneum levels (*P* < 0.001). As surgical time increased, the observed group had a smaller effect on the deep venous blood flow dynamics of the lower limbs than the control group. The deep femoral vein diameter in the observed group was thinner than the control group, while the mean blood flow velocity and mean blood flow rate were increased, with statistically significant differences (*P* < 0.05). Compared to the previous position angle, there were statistically significant changes in deep femoral vein blood flow dynamics in the observed group at T2, T3, and T6 (*P* < 0.001), and in the control group at T2, T3, T4, and T6 (*P* < 0.001). Compared to T0, there were statistically significant differences in deep femoral vein diameter, mean blood flow rate at T2, T3, T4, and T5, and mean blood flow velocity at T2, T3, T4, T5, and T6 in the observed group (*P* < 0.001), and in the control group for mean blood flow rate at T2, T3, T4, and T5, and deep femoral vein diameter and mean blood flow velocity at T2, T3, T4, T5, and T6 (*P* < 0.001).

### Comparison of deep vein ultrasound examination before leaving the operating room in two groups

As shown in Table 2. In both groups, anesthesia induction ultrasound examination showed unobstructed blood flow in both lower limbs. Before leaving the operating room, no DVT occurred in either group, but one case of lower limb venous stasis (1.02%) was observed in the observation group, while 11 cases of lower limb venous stasis (11.22%) were found in the control group, all of which occurred in a single lower limb. The venous blood flow in the lower limbs of the observation group was significantly better than that in the control group, and the difference was statistically significant (*P*=0.003).

**Table 2:** Test results of deep venous ultrasonography in both lower limbs of patients in the two groups before leaving the operating room after the surgery (cases, %)

Group	Blood flow unobstruction	Blood flow stagnation	DVT
Observation group(*n*=98)	97(98.98)	1(1.02)	0
Control group(*n*=98)	87(88.78)	11(1.22)	0
Z value	8.877		
*P* value	0.003		

### Results of the intraoperative course of each patient in the two groups

Compared in Table 3. In both groups, the operative times of patients were nearly 150mins, and the difference was not statistically significant (*p*=0.261). Similarly, the difference in blood loss was also not statistically significant (*p*=0.247), blood loss of patients in both groups was averaged 150ml. As to complications rates, there were no serious complications such as death or real urinary incontinence happened in each group. As shown in Table 4, the incidence of DVT in the control group was higher than that in the observation group, and the difference was statistically significant (*P* =0.041).

**Table 3:** The intraoperative course of each individual patient

Group	Operative times[min, M(P25, P75)]	Blood loss[ml, M(P25, P75)]
Observation group (*n*=98)	148.00(126.75,182.25)	150.00(70.00,215.00)
Control group (*n*=98)	153.00(132.50,186.25)	150.0(70.00,246.25)
*Z* value	1.125	0.805
*P* value	0.261	0.247

**Table 4:** Postoperative complications in both groups (cases, %)

group	nausea and vomiting	pneumonia	urinary extravasation	urinary incontinence	intestinal adhesion	DVT	PTE	total
Observation group (*n*=98)	4(4.1)	2(2.0)	6(6.1)	2(2.0)	3(1.0)	1(1.0)	0	18(18.4)
Control group (*n*=98)	6(6.1)	3(3.1)	5(5.1)	3(3.1)	2(5.1)	8(8.2)	0	27(27.6)
*χ*^*2*^value	0.422	0.000	0.096	0.000	0.000	4.193	-	2.336
*P* value	0.516^(1)^	1.000^(2)^	0.756^(1)^	1.000^(2)^	1.000^(2)^	0.041^(2)^	-	0.126^(1)^

(1) Pearson χ2 test

(2) continuity corrected χ2 test

## Discussion

Stasis of venous blood flow, blood hypercoagulability, and venous wall injury are the three elements that contribute to the formation of deep vein thrombosis (DVT) [5]. DVT occurring in the lower extremities is one of the main complications that need to be prevented and treated during the perioperative period, owing to its high incidence of disability and mortality [6]. The risk of DVT in lower limbs in postoperative patients can be as high as 20%-70% [9]. Early and effective prevention can reduce the occurrence of DVT by 30%-80% [10, 11], among which basic prevention is the fundamental and effective approach to prevent DVT [12].

**Fig. 5:**
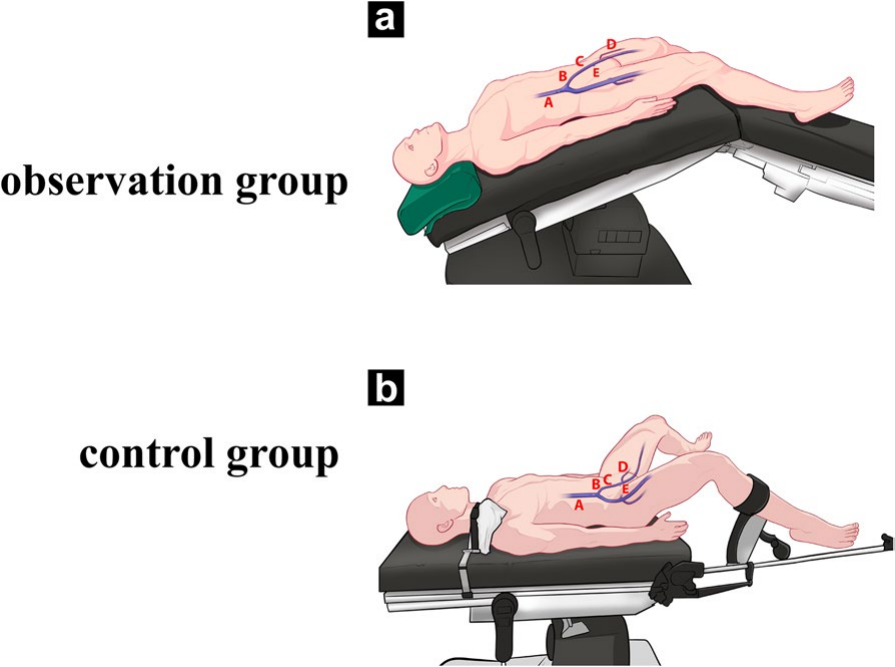
Distribution and morphology of some veins in two positions. **a** observation group; **b** control group; **A** inferior vena cava; **B** external iliac vein; **C** common femoral vein; **D** deep femoral vein; **E** great saphenous vein

This study found that in both groups, after pneumoperitoneum was established, the diameter of the deep femoral vein significantly increased, and the mean blood flow velocity and mean blood flow significantly decreased compared to before pneumoperitoneum, with significant differences in both groups (*P*<0.001), which is consistent with the study by Wang et al. [13]. The normal pressure of the inferior vena cava is 2-5 mmHg, and the pneumoperitoneum pressure for laparoscopic surgery needs to be maintained at 12-14 mmHg and not exceed 15 mmHg. The increased intra-abdominal pressure directly compresses the inferior vena cava and bilateral iliac veins, hindering blood flow and resulting in increased deep femoral vein diameter. With prolonged surgical time, the observed group had less impact on the deep venous hemodynamics of the lower limbs compared to the control group. The deep femoral vein diameter of the observed group at T2-T6 was smaller than that of the control group, and the mean blood flow velocity and mean blood flow increased, with significant differences in both groups (*P*<0.05). This may be related to the fact that the control group formed an angle at the hip joint during low lithotomy position (Fig. 5b), and the pneumoperitoneum pressure and body position angle pulled or even squeezed the common femoral vein, resulting in a thinner common femoral vein diameter, slower blood flow velocity, reduced blood flow, hindered blood flow reflux, blood stasis, and subsequently deep femoral vein diameter expansion, which may cause micro-tears in the blood vessels and expose collagen fibers [14], providing conditions for the formation of lower limb venous thrombosis. In the observed group, the thigh was kept horizontal in a single-leg position, and the hip joint was in a physiological position, avoiding the formation of an angle at the thigh that could cause traction on the hip joint and thigh muscles and bones. The legs of the observed group were well supported (Fig. 5a), dispersing the weight of the legs and avoiding the disadvantage of direct compression of the popliteal fossa in the control group and the calf muscles supporting the weight of the legs, improving the venous blood flow in the leg and reducing the intravascular pressure. The distal end of the lower limbs of the observed group, i.e., the toes, was at the same horizontal level as the heart, avoiding the influence of gravity on the venous blood flow in the deep veins of the lower limbs [15]. Compared with the previous body position angle, the changes in deep femoral vein hemodynamics at T3 and T6 were statistically significant in the observed group (*P*<0.001), and the changes in deep femoral vein hemodynamics at T3, T4, and T6 were statistically significant in the control group (*P*<0.001). This indicates that the effect of gravity is conducive to the venous blood flow in the lower limbs and to some extent relieves the venous blood stasis caused by pneumoperitoneum. On the other hand, it suggests that the duration of pneumoperitoneum is positively correlated with the increase in deep femoral vein diameter and the decrease in mean blood flow velocity and mean blood flow, and minimizing the traction and compression of blood vessels caused by body position angles can alleviate the deep venous blood flow stasis caused by pneumoperitoneum. As the median surgical times of the two groups in this study were 148.00 h and 153.00 h, respectively, T5 was close to T4 in time, so the changes in blood flow dynamics in T5 of the two groups were not statistically significant compared to T4.

When positioning a robot for surgery, it is important to maintain the normal physiological curvature and axis of the human body, as well as the physiological functional position of each limb and joint, in order to prevent excessive traction, torsion, and damage to blood vessels and nerves [16]. This also cannot obstruct the robot's access or the movement of the observation mechanical arm, while meeting the surgeon's surgical requirements and not causing damage to the patient or the mechanical arm. This is both a requirement for the positioning of the robot for surgery and one of the basic preventive measures for preventing DVT. In conclusion, this study illustrates that placing patients in the Trendelenburg position and leg split position with calf reverse arch during RARP has a smaller impact on lower extremity hemodynamics than the lithotomy position, and can relatively reduce the risk of postoperative deep vein thrombosis. The position could be promoted and applied in clinic. It has been shown that the hemodynamics may be related to patients’ metabolic risk factors (body weight, abdominal girth, BP, et al) [17]. The reason is that patient's metabolic health affects their basic diseases (high blood pressure, hyperlipidemia, diabetes, et al), which in turn affects the patient’s hemodynamics and blood properties. In addition, Trendelenburg position and general anesthesia may induce autonomic changes and thus hemodynamic changes, even more severe bradycardia and cardiac arres [18]. So the effect of these factors on lower extremity hemodynamics cannot be ignored. It is an interesting topic worth exploring in next study, these factors will be considered in addition to patients’ positions to make the results more convincing and find a comprehensive program during operations to reduce the incidence of DVT. The shortcomings of this study include being conducted at a single center and having a small number of cases. Furthermore, there was no further monitoring of the postoperative hemodynamics of the patients. Therefore, further large-scale and improved clinical studies are needed to confirm the conclusions of this study. The next step for this research team is to collaborate with multiple centers and further expand the sample size to provide more sufficient data support and clinical application information for the supine position with head lower than feet, legs spread and calf hanging down in robot-assisted laparoscopic urological surgery.

## Data Availability

The data that support the findings of this study can be available from the corresponding author on reasonable request.
